# Exploring Senior Dental Students’ Readiness and Perceptions of Artificial Intelligence in Dentistry: Insights From a Cross‐Sectional Study

**DOI:** 10.1155/ijod/8981807

**Published:** 2026-08-14

**Authors:** Nasreen Hamarash Hamonari

**Affiliations:** ^1^ Dental Public Health Program, Kurdistan Higher Council of Medical Specialties, Erbil, Kurdistan Region, Iraq

**Keywords:** artificial intelligence, attitude, cross-sectional studies, dental, dentistry, students

## Abstract

**Background:**

Artificial intelligence (AI) is increasingly influencing dentistry; however, senior dental students’ readiness to understand and use AI remains insufficiently explored in underrepresented settings. This study assessed knowledge, perceptions, attitudes, and readiness toward AI among senior dental students in Erbil, Kurdistan Region of Iraq.

**Methods:**

A cross‐sectional survey was conducted among 168 senior dental students using an expert‐reviewed and pilot‐tested questionnaire adapted from previously used instruments. AI‐related readiness was assessed using a descriptive knowledge/use index and two normalized composite scores: perception/clinical‐application and attitude/future‐expectation scores. Internal consistency was assessed for the two multi‐item composite scores.

**Results:**

Self‐rated AI knowledge was moderate in 45.2% of students and high in 11.9%, while prior involvement in AI‐related development or projects was limited (5.4%). The knowledge/use index was low (0.25 ± 0.20), indicating limited practical exposure and familiarity. In contrast, the perception/clinical‐application score was high (0.69 ± 0.16), with favorable views particularly toward implant planning, radiographic caries detection, quality control, and diagnosis and treatment planning. The attitude/future‐expectation score was also high (0.69 ± 0.26), indicating positive expectations regarding the future role of AI in dentistry. The perception/clinical‐application score showed acceptable internal consistency (Cronbach’s alpha = 0.78), while the attitude/future‐expectation score showed high internal consistency (Cronbach’s alpha = 0.86). No statistically significant differences were observed in knowledge/use, perception/clinical‐application, or attitude/future‐expectation scores according to gender or year of study.

**Conclusion:**

Senior dental students demonstrated favorable perceptions and expectations toward AI in dentistry, particularly for diagnostic and treatment‐related applications. However, practical exposure and AI‐related use were limited, and concerns remained regarding empathy, accountability, ethical issues, and human oversight. Because this was an Erbil‐based convenience sample, the findings should be interpreted cautiously and may not generalize to all dental students.

## 1. Introduction

Artificial intelligence (AI) is increasingly transforming healthcare by supporting clinical decision‐making, improving diagnostic accuracy, enhancing workflow efficiency, and assisting in the interpretation of large and complex datasets [1, 2]. In response to expanding clinical demands and the growing availability of digital health data, AI‐based systems are being introduced across several medical specialties to support diagnosis, risk prediction, treatment planning, and patient monitoring [3, 4]. These systems use approaches such as machine learning, deep learning, and neural networks to recognize patterns, process complex information, and generate outputs that may assist clinicians in tasks traditionally dependent on human judgment [5, 6].

In dentistry, AI has gained increasing attention because of its potential to support both clinical and educational practice. AI‐enabled virtual assistants, chatbots, and teledentistry platforms may improve patient education, communication, and remote consultation, particularly when integrated with clinical records and patient‐specific data [7]. AI applications can also assist in radiographic interpretation, caries detection, periodontal assessment, cephalometric analysis, oral cancer screening, implant planning, prosthetic design, and personalized treatment planning [8–11]. These developments suggest that future dentists will increasingly encounter AI‐supported tools in the diagnostic, preventive, therapeutic, and administrative aspects of dental care.

Despite these advances, the routine integration of AI into dental practice remains limited. Several challenges continue to affect its adoption, including limited access to high‐quality datasets, concerns regarding model generalizability, algorithmic transparency, data privacy, legal accountability, and professional responsibility for AI‐assisted decisions [12, 13]. In addition, dentistry remains a highly interpersonal profession in which communication, empathy, clinical judgment, and patient trust are essential. Therefore, AI is more appropriately viewed as a supportive tool rather than a replacement for the dentist, particularly in situations requiring ethical reasoning, individualized care, and management of unexpected clinical circumstances [7, 14, 15].

Dental students represent an important group for evaluating AI readiness because they are future clinicians who will be expected to use, evaluate, and critically interpret AI‐supported technologies. Previous studies from different countries have shown that dental students generally express interest in AI and recognize its potential usefulness in diagnosis, radiology, treatment planning, and dental education [16–18]. However, these studies have also reported variable levels of knowledge, practical exposure, confidence, and concern regarding ethical, legal, and professional implications [19–22]. Recent evidence further suggests that students’ acceptance of AI may be influenced by educational exposure, institutional resources, professional seniority, digital confidence, and perceived relevance to clinical practice [23–27]. Thus, although the literature on dental students’ perceptions of AI is growing, the findings remain context‐dependent and cannot be generalized across all educational systems.

Evidence from Iraq and the Kurdistan Region remains scarce. This gap is important because students’ readiness for AI may be shaped by local curriculum design, clinical training exposure, digital infrastructure, faculty expertise, and access to AI‐related educational resources. Erbil, located in the Kurdistan Region of Iraq, represents a rapidly expanding academic and clinical environment. However, structured AI training and formal curricular integration remain limited, and institutions may face constraints related to infrastructure, faculty expertise, and access to advanced technologies. These contextual factors justify the need for localized investigation.

The present study aimed to explore senior dental students’ readiness and perceptions toward AI in dentistry in Erbil City, Kurdistan Region of Iraq. The main research question was as follows: What are senior dental students’ levels of readiness toward AI applications in dentistry? The null hypothesis stated that there would be no statistically significant differences in normalized knowledge/use, perception/clinical‐application, and attitude/future‐expectation scores according to gender or year of study.

## 2. Materials and Methods

### 2.1. Study Design and Participants

A cross‐sectional, questionnaire‐based study was conducted among undergraduate dental students in Erbil City. Fourth‐ and fifth‐year dental students were invited to participate because they had sufficient clinical exposure to provide informed opinions on the potential role of AI in dentistry. Participation was voluntary and anonymous. Students who agreed to participate and submitted fully completed questionnaires were included. Questionnaires with incomplete or incorrectly completed responses were excluded from the final analysis.

### 2.2. Ethical Considerations

Ethical approval was obtained from the institution’s Clinical Research Ethics Committee (Approval Number 1558; May 5, 2025). Participation was voluntary and anonymous. Confidentiality of the collected data was maintained throughout the study, and access to the dataset was limited to the principal investigator.

### 2.3. Sampling Strategy and Sample Size

A convenience sampling approach was used. The required sample size was estimated for a single proportion using a 90% confidence level, a 5% margin of error, and an assumed proportion of 50%, which provides the maximum required sample size when the expected prevalence is unknown. After applying finite population correction for an estimated eligible population of ~400 students, the minimum required sample size was 162 completed responses. To compensate for incomplete or incorrectly completed questionnaires, 230 students were invited to participate.

The 90% confidence level was considered acceptable because the study was exploratory and conducted within a limited, clearly defined student population. The use of convenience sampling may limit generalizability and introduce selection bias; however, students from both eligible academic years were invited to improve representation within the available study population.

### 2.4. Questionnaire Development and Validation

Data were collected using a structured self‐administered questionnaire developed after reviewing relevant literature and adapted from previously used instruments on AI awareness, perceptions, and attitudes in healthcare and dentistry [18, 20, 24–28]. The questionnaire included sections on demographic characteristics, AI knowledge and use, perceived advantages and disadvantages of AI, perceptions of AI in dental education and diagnosis, clinical applications of AI, responsibility and trust in AI‐assisted decision‐making, and expectations regarding the future role of AI in dentistry.

The questionnaire was reviewed for face and content validity by experts in dentistry and dental public health. Items were assessed for clarity, relevance, and appropriateness for undergraduate dental students. A pilot assessment was conducted among 15 senior dental students before data collection to evaluate the clarity, comprehensibility, and appropriateness of the questionnaire items and response options. Based on pilot feedback, minor wording modifications were made where necessary. Pilot responses were not included in the final analysis.

### 2.5. Outcome Measures and Composite Score Construction

The primary outcome was students’ readiness toward AI in dentistry, assessed using three analytic indices: a knowledge/use index, a perception/clinical‐application score, and an attitude/future‐expectation score. In this study, readiness was operationally defined as students’ level of AI‐related knowledge and use, perceived acceptance of AI clinical applications, and attitudes toward the future role of AI in dentistry.

The knowledge/use index was based on three indicators: self‐rated AI knowledge, previous involvement in AI‐related development or projects, and frequency of AI use. These variables represent related but distinct aspects of AI exposure rather than repeated measures of a single latent construct; therefore, this index was interpreted descriptively, and Cronbach’s alpha was not calculated for it.

The perception/clinical‐application score was based on seven direct diagnostic and treatment‐related AI application items: AI support for diagnosis and treatment planning, radiographic diagnosis of caries, diagnosis of oral soft‐tissue lesions, identification of jaw pathologies, detection of periodontal diseases, applications in forensic odontology, and 3D implant positioning and planning. Items related to prognosis and quality control were reported descriptively but were not included in this composite score because they represent broader supportive functions rather than direct diagnostic or treatment‐planning applications.

The attitude/future‐expectation score was based on four items assessing expectations toward the future impact of AI in dentistry: AI leading to great advances in dentistry, AI improving clinical practice, AI reducing medical errors, and AI reducing workforce demand.

All composite scores were normalized to a scale from 0 to 1. Binary items were scored as 1 for “Yes” and 0 for “No.” AI use frequency was scored as 1 for daily use, 0.5 for weekly use, and 0 for rare or no use. For three‐category items, responses were scored as 1 for favorable/high responses, 0.5 for neutral/moderate responses, and 0 for unfavorable/low responses. For five‐point Likert items, responses were scored as 1 for strongly agree, 0.75 for agree, 0.50 for neither agree nor disagree, 0.25 for disagree, and 0 for strongly disagree. For the workforce reduction item, responses were scored as 1 for significant reduction, 0.5 for moderate reduction, and 0 for little or no reduction. Composite scores were calculated as the mean of the included recoded items.

For interpretation, normalized scores from 0.00 to 0.33 were considered low, scores > 0.33 to 0.66 were considered moderate, and scores > 0.66 to 1.00 were considered high, favorable, or indicative of stronger expectation, depending on the construct being assessed.

### 2.6. Statistical Analysis

Data were analyzed using SPSS software. Descriptive statistics were presented as frequencies and percentages for categorical variables and as means, standard deviations, medians, and ranges for normalized composite scores. Internal consistency was assessed using Cronbach’s alpha for the multi‐item perception/clinical‐application and attitude/future‐expectation scores. Cronbach’s alpha was not calculated for the knowledge/use index because it consisted of heterogeneous exposure‐related indicators rather than parallel scale items.

Independent‐samples *t*‐tests were used to compare normalized composite scores according to gender and year of study, which was treated as a prespecified subgroup variable. Mean differences were reported with 95% confidence intervals. Cohen’s *d* was calculated as a measure of effect size. Statistical significance was set at *p* < 0.05.

## 3. Results

### 3.1. Demographics

A total of 230 students were recruited. Of these, 62 questionnaires were excluded because they were incomplete or incorrectly completed, leaving 168 fully completed questionnaires for final analysis and giving a valid response rate of 73.0%. Females comprised 54.2% of participants (*n* = 91), while males comprised 45.8% (*n* = 77). Most participants were in the fourth year of study (54.2%, *n* = 91), followed by the fifth year (45.8%, *n* = 77).

### 3.2. AI Knowledge and Use

Self‐rated AI knowledge was most commonly reported as moderate (45.2%, *n* = 76), followed by low (42.9%, *n* = 72) and high (11.9%, *n* = 20). Only 5.4% of students (*n* = 9) reported previous involvement in AI‐related development or projects. Regarding AI use frequency, 16.1% reported daily use, 36.9% reported weekly use, and 47.0% reported rare or no use (Table 1).

**Table 1:** Demographic characteristics and AI experience of participants (*n* = 168).

Variable	Category	*n*	%
Gender	Male	77	45.8
	Female	91	54.2
Year of study	4th year	91	54.2
	5th year	77	45.8
AI knowledge	High	20	11.9
	Moderate	76	45.2
	Low	72	42.9
Previous involvement in AI‐related development/projects	Yes	9	5.4
	No	159	94.6
AI use frequency	Daily	27	16.1
	Weekly	62	36.9
	Rarely/not at all	79	47.0

*Note:* Values are presented as frequency (*n*) and percentage (%).

### 3.3. Perceptions of AI

Students most frequently identified fast and objective outcomes (39.9%) and integration of large amounts of data (31.5%) as the main advantages of AI in dentistry. Limited empathy (32.7%) and difficulty handling unexpected cases (24.4%) were the most frequently reported disadvantages. Most students supported the integration of AI into dental curricula (63.7%) and considered AI essential in dental practice (73.8%). A high proportion agreed that AI can assist in early diagnosis and disease severity assessment (79.2%), whereas only 10.7% agreed that AI could outperform experienced dental professionals. When AI and dentists’ judgments disagreed, 50.6% trusted the dentist’s judgment, and 67.3% assigned responsibility for AI‐related misdiagnosis to the dentist (Table 2).

**Table 2:** Perceived advantages, disadvantages, and responsibilities of AI in dentistry.

Question	Response category	*n*	%
The main advantage of AI in dentistry	Fast and objective outcomes	67	39.9
	Integration of vast data	53	31.5
	Reduction of misdiagnosis rates	26	15.5
	No spatial/temporal constraints	11	6.5
	No emotional/physical limitations	8	4.8
	Other	3	1.8
Major disadvantage of AI in dentistry	Limited empathy	55	32.7
	Difficulty handling unexpected cases	41	24.4
	Difficulty with controversial issues	32	19.0
	Somewhat inflexible for individual patients	29	17.3
	Developed by experts with limited clinical experience	9	5.4
	Other	2	1.2
AI integration into dental curricula	Agree/strongly agree	107	63.7
	Neither agree nor disagree	46	27.4
	Disagree/strongly disagree	15	8.9
AI is essential in dental practice	Agree/strongly agree	124	73.8
	Neither agree nor disagree	32	19.0
	Disagree/strongly disagree	12	7.1
AI aids early diagnosis and severity assessment	Agree/strongly agree	133	79.2
	Neither agree nor disagree	26	15.5
	Disagree/strongly disagree	9	5.4
AI outperforms expert diagnostics	Agree/strongly agree	18	10.7
	Neither agree nor disagree	58	34.5
	Disagree/strongly disagree	92	54.8
Who would you trust more if AI and a dentist disagree?	Dentist’s judgment	85	50.6
	Opinions of other experts	61	36.3
	Leave it to the patient’s choice	14	8.3
	AI’s judgment	4	2.4
	Opinions of other AI programs	4	2.4
Responsibility when AI misdiagnoses	Dentist	113	67.3
	AI developer company	38	22.6
	Patient who followed AI	13	7.7
	Dental hygienist	3	1.8
	Other	1	0.6

*Note:* Values are presented as frequency (*n*) and percentage (%).

### 3.4. Clinical Applications of AI

High levels of agreement were observed for AI use in 3D implant planning (85.7%), radiographic diagnosis of caries (83.9%), and quality control (82.1%). Agreement was also high for diagnosis and treatment planning (73.2%), periodontal disease detection (73.2%), and soft‐tissue lesion diagnosis (71.4%). Moderate endorsement was observed for prognosis (66.7%), forensic odontology applications (60.1%), and jaw pathology identification (58.3%) (Table 3).

**Table 3:** Clinical applications and perceived effectiveness of AI.

Question	Agree, *n* (%)	Neutral, *n* (%)	Disagree, *n* (%)
AI can be used as a prognostic tool	112 (66.7)	36 (21.4)	20 (11.9)
AI supports diagnosis and treatment planning	123 (73.2)	16 (9.5)	29 (17.3)
AI for radiographic diagnosis of caries	141 (83.9)	13 (7.7)	14 (8.3)
AI diagnosis of oral soft‐tissue lesions	120 (71.4)	28 (16.7)	20 (11.9)
AI helps identify jaw pathologies	98 (58.3)	37 (22.0)	33 (19.6)
AI can detect periodontal diseases	123 (73.2)	29 (17.3)	16 (9.5)
AI for 3D implant planning	144 (85.7)	11 (6.5)	13 (7.7)
AI applications in forensic odontology	101 (60.1)	38 (22.6)	29 (17.3)
AI is effective as a quality control tool	138 (82.1)	14 (8.3)	16 (9.5)

*Note:* Agree includes strongly agree/agree; disagree includes disagree/strongly disagree.

### 3.5. Expectations and Attitudes

Two‐thirds of students agreed that AI will lead to major advances in dentistry (66.7%), whereas 28.0% believed that AI may replace dentists. Nearly half expected AI to be used in most practices once applied (46.4%). Most participants expected AI to noticeably impact dentistry within 6–10 years (38.7%) or after more than 10 years (35.1%). Students also expected AI to improve clinical practice (64.3%) and reduce medical errors (78.0%). Regarding workforce implications, 57.7% predicted a moderate reduction in demand, 20.8% predicted a significant reduction, and 21.4% predicted little or no reduction. Regarding the expected role of AI, the most common responses were that AI would complement the limits of human intelligence (36.3%), serve as a reference for each treatment (29.8%), or provide clinical data for evidence‐based dentistry (20.8%) (Table 4).

**Table 4:** Expectations and attitudes toward AI.

Question	Response category	*n*	%
AI will lead to great advances in dentistry	Agree/strongly agree	112	66.7
	Neither agree nor disagree	27	16.1
	Disagree/strongly disagree	29	17.3
AI may replace dentists	Agree/strongly agree	47	28.0
	Neither agree nor disagree	17	10.1
	Disagree/strongly disagree	104	61.9
How often will AI be used once applied	Most practices	78	46.4
	Half the time	34	20.2
	Only when necessary	56	33.3
When will AI noticeably impact the profession?	Within 1 year	3	1.8
	In 1–5 years	34	20.2
	In 6–10 years	65	38.7
	After 10 years	59	35.1
	Never	7	4.2
AI can improve clinical practice	Agree/strongly agree	108	64.3
	Neither agree nor disagree	40	23.8
	Disagree/strongly disagree	20	11.9
AI will reduce medical errors	Agree/strongly agree	131	78.0
	Neither agree nor disagree	24	14.3
	Disagree/strongly disagree	13	7.7
Extent AI will reduce the workforce	Significant reduction	35	20.8
	Moderate reduction	97	57.7
	Little/none	36	21.4
Expected role of AI in dental healthcare	Complement human intelligence limits	61	36.3
	Reference for each treatment	50	29.8
	Provide clinical data for evidence‐based dentistry	35	20.8
	Guide for rare cases	17	10.1
	Not helpful for dental healthcare	2	1.2
	Other	3	1.8

*Note:* Values are presented as frequency (*n*) and percentage (%).

### 3.6. Composite Scores and Internal Consistency

The normalized composite scores are presented in Table 5. Scores from 0.00 to 0.33 were interpreted as low, scores > 0.33 to 0.66 as moderate, and scores > 0.66 to 1.00 as high or favorable. The mean knowledge/use index was low (0.25 ± 0.20), indicating limited AI‐related exposure and familiarity among participants. In contrast, the perception/clinical‐application score was high (0.69 ± 0.16), reflecting generally favorable views toward direct clinical applications of AI. The attitude/future‐expectation score was also high (0.69 ± 0.26), indicating strong expectations regarding the future impact of AI in dentistry.

**Table 5:** Internal consistency and normalized composite scores.

Composite score	Number of items	Cronbach’s alpha	Mean ± SD	Median	Min–max	Interpretation
Knowledge/use index	3	Not applicable	0.25 ± 0.20	0.17	0.00–1.00	Low
Perception/clinical‐application score	7	0.78	0.69 ± 0.16	0.71	0.21–1.00	High
Attitude/future‐expectation score	4	0.86	0.69 ± 0.26	0.81	0.00–1.00	High

*Note:* Composite scores were normalized from 0 to 1. The knowledge/use index was interpreted as a formative descriptive index. The perception/clinical‐application score included seven direct diagnostic and treatment‐related AI application items. The attitude/future‐expectation score included AI advances, improvement of clinical practice, reduction of medical errors, and expected workforce reduction.

Cronbach’s alpha did not apply to the knowledge/use index because it was a formative index based on heterogeneous exposure‐related indicators. The perception/clinical‐application score demonstrated acceptable internal consistency (Cronbach’s alpha = 0.78), while the attitude/future‐expectation score demonstrated high internal consistency (Cronbach’s alpha = 0.86).

### 3.7. Gender and Year‐of‐Study Comparisons

No statistically significant differences in knowledge/use, perception/clinical‐application, or attitude/future‐expectation scores were observed according to gender or year of study. Mean differences were small, all 95% confidence intervals crossed zero, and Cohen’s *d* values indicated small effect sizes across all comparisons (Table 6).

**Table 6:** Comparison of composite scores according to gender and year of study.

Variable	Comparison	Group 1 (mean ± SD)	Group 2 (mean ± SD)	Mean difference (95% CI)	Cohen’s *d*	*p*‐Value
Knowledge/use index	Male vs. female	0.27 ± 0.22	0.23 ± 0.18	0.04 (−0.03 to 0.10)	0.19	0.240
Perception/clinical‐application score	Male vs Female	0.70 ± 0.18	0.68 ± 0.15	0.03 (−0.02 to 0.08)	0.17	0.278
Attitude/future‐expectation score	Male vs. female	0.70 ± 0.28	0.68 ± 0.25	0.02 (−0.06 to 0.10)	0.07	0.644
Knowledge/use index	4th year vs. 5th year	0.26 ± 0.20	0.24 ± 0.21	0.02 (−0.04 to 0.08)	0.11	0.481
Perception/clinical‐application score	4th year vs. 5th year	0.69 ± 0.17	0.69 ± 0.16	0.00 (−0.04 to 0.05)	0.03	0.853
Attitude/future‐expectation score	4th year vs. 5th year	0.70 ± 0.26	0.68 ± 0.27	0.02 (−0.06 to 0.10)	0.08	0.597

*Note:* Mean difference represents Group 1 minus Group 2. Cohen’s *d* was used as the effect size. Statistical significance was set at *p* < 0.05.

Abbreviation: CI, confidence interval.

## 4. Discussion

The present study evaluated senior dental students’ readiness and perceptions toward AI in dentistry in Erbil City. The findings demonstrate a clear contrast between students’ limited practical exposure to AI and their generally favorable perceptions of its potential clinical value. This pattern was reflected in the predefined normalized composite scores, where the knowledge/use index was low, while the perception/clinical‐application and attitude/future‐expectation scores were high. These findings suggest that students are receptive to AI and recognize its potential relevance to dentistry, but their actual familiarity, direct use, and practical preparedness remain limited. Therefore, the findings suggest that the main challenge is not apparent resistance to AI but rather the gap between favorable attitudes and limited practical readiness.

The low knowledge/use index is particularly important. Although a considerable proportion of students rated their AI knowledge as moderate, direct experience with AI‐related tools or projects was uncommon, and nearly half reported rare or no AI use. This suggests that students’ understanding of AI may be based mainly on general awareness rather than structured education or hands‐on experience with AI‐supported tools. Similar gaps have been reported in studies among dental students and professionals, where favorable attitudes toward AI were not always matched by adequate knowledge, confidence, training, or practical use [16, 17, 19, 24, 28–30]. This finding is also consistent with broader evidence showing that AI readiness in dental education is influenced by educational exposure, curriculum integration, access to training, and perceived relevance to clinical practice [19, 30–34]. In the present context, the low knowledge/use score may reflect the limited formal integration of AI into dental curricula and the lack of structured opportunities for students to interact with AI‐based clinical or educational systems.

In contrast, the high perception/clinical‐application score indicates that students held favorable views toward AI when it was linked to specific clinical tasks. Agreement was particularly high for AI use in radiographic caries detection, implant planning, diagnosis and treatment planning, periodontal disease detection, and quality control. These applications rely heavily on image interpretation, pattern recognition, data processing, and standardization, which may explain why students perceived AI as useful in these areas. Previous studies and reviews have similarly reported that AI is increasingly viewed as valuable in dental radiology, restorative dentistry, oral medicine, orthodontic treatment planning, implantology, forensic odontology, and digital workflow applications [20, 21, 26, 35–40]. Hegde et al. [20] also reported that dentists and dental students recognized the potential of AI in dentomaxillofacial radiology and implantology, although many current AI systems remain task‐specific rather than broadly applicable across all areas of dentistry. These findings suggest that students may be more willing to accept AI when it is presented as a supportive tool for objective, data‐driven, and technically oriented clinical tasks.

The high attitude/future‐expectation score further indicates optimism regarding the future role of AI in dentistry. Many participants expected AI to contribute to advances in dental practice, improve clinical performance, and reduce errors. However, this optimism was accompanied by caution regarding workforce replacement. Most students did not view AI as a substitute for dentists but rather as a tool that may support diagnosis, treatment planning, workflow efficiency, and quality assurance. This interpretation is consistent with studies and reviews involving dental students and dental education, where AI is generally viewed positively but concerns remain regarding clinical judgment, ethical use, educational preparedness, and responsible integration into dental curricula [18, 22, 26, 28, 29, 32, 41, 42]. Thus, the findings suggest cautious optimism rather than uncritical acceptance of AI.

Students’ concerns regarding empathy, unexpected clinical situations, ethical dilemmas, and accountability are also clinically meaningful. Dentistry is not limited to diagnosis and technical decision‐making; it also requires communication, empathy, patient trust, ethical reasoning, and individualized judgment. AI may be useful for analyzing images, organizing data, and supporting decision‐making, but it cannot independently address the full human and contextual dimensions of dental care. Previous literature has emphasized that although AI may automate selected tasks such as image analysis and data processing, dental care remains a patient‐centered profession requiring professional judgment, clinical experience, interpersonal communication, and consideration of patients’ preferences and circumstances [43, 44]. Therefore, AI is more likely to support and reshape selected aspects of dental practice than to replace dentists entirely.

The findings related to trust and responsibility further support this interpretation. A large proportion of students assigned responsibility for AI‐related misdiagnosis to the dentist, and many preferred the dentist’s judgment when AI and clinical opinions disagreed. This suggests that students recognized the continuing responsibility of the clinician in AI‐assisted care. This is important because AI implementation in dentistry raises ethical and legal questions related to accountability, transparency, privacy, bias, data protection, informed consent, and professional responsibility [12, 13, 16, 27, 45]. Therefore, AI education should not focus only on technical functions. It should also include ethical reasoning, medico‐legal responsibility, patient safety, critical appraisal of AI outputs, and appropriate human oversight.

No statistically significant differences were observed in knowledge/use, perception/clinical‐application, or attitude/future‐expectation scores according to gender or year of study. The mean differences were small, confidence intervals crossed zero, and Cohen’s *d* values indicated small effect sizes. This suggests that male and female students, as well as fourth‐ and fifth‐year students, had broadly similar readiness toward AI in this sample. Similar findings were reported by Elchaghaby and Wahby and Ezzeldin et al., who found no significant gender‐based differences in AI‐related knowledge, perception, or attitude among dental students [17, 24]. This finding is also partly consistent with Yazdi et al., who reported that demographic factors were not significantly associated with AI readiness among faculty members, although findings among student groups may vary [33]. However, other studies have reported demographic or gender‐related differences in AI readiness and ethical concerns [30, 46]. These inconsistencies suggest that gender differences in AI readiness may be context‐dependent and influenced by educational exposure, digital experience, training opportunities, and professional environment rather than gender alone.

The absence of significant differences according to the year of study is also informative. Although fifth‐year students generally have greater clinical exposure than fourth‐year students, this did not translate into higher AI knowledge, perception, or attitude scores in the present study. One possible explanation is that both academic years were exposed to similar curricula and institutional learning environments, with limited formal AI‐related training. This finding suggests that general progression through clinical years may not be sufficient to improve AI readiness unless AI is deliberately incorporated into dental education. Therefore, improvements in AI readiness may require structured curricular content, practical demonstrations, and guided exposure to AI‐supported diagnostic and educational tools.

The present findings are consistent with recent evidence on AI implementation and digital transformation in dentistry. Studies among postgraduate dental students, dental faculty members, dentists, and dental professionals have generally reported positive perceptions of AI while also identifying concerns regarding accuracy, reliability, ethics, training, cost, implementation, and the need for human supervision [46–53]. Sarhan et al. [49] reported generally positive perceptions among postgraduate dental students but also identified concerns related to clinical reliability and responsible implementation. Abdullah et al. [51] found that dental faculty members supported AI integration, although limited training opportunities, financial constraints, and ethical concerns were important barriers. Similarly, Yazdi et al. emphasized the need for AI‐focused education to improve theoretical and practical competencies among dental faculty members and students [33]. Altındağ et al. also reported that dental students and dentists recognized the educational relevance of AI, but gaps remained in knowledge, preparedness, and formal AI‐related training [30]. Together, these studies support the present finding that positive attitudes alone are not enough; responsible AI adoption requires knowledge, practical competence, institutional readiness, and ethical guidance.

The findings have important implications for dental education. As AI applications continue to expand, dental education should move beyond informal awareness toward structured AI literacy. Schwendicke et al. [31] proposed a core AI curriculum for oral and dental healthcare, emphasizing foundational AI concepts, clinical use cases, evaluation metrics, explainability, accountability, governance, and ethical considerations. Recent scoping reviews have also highlighted the importance of integrating AI into dental education through structured strategies that address student and educator preparedness, practical applications, ethical and legal responsibilities, and implementation gaps [32, 34]. In this context, AI education should not be presented as purely technical training. It should prepare students to understand the strengths and limitations of AI systems, critically evaluate AI‐generated recommendations, recognize bias and uncertainty, protect patient data, and maintain professional accountability.

Overall, this study shows that senior dental students in Erbil demonstrate cautious optimism toward AI in dentistry. They recognize its potential to support diagnostic accuracy, treatment planning, implant planning, workflow efficiency, and quality control while also acknowledging limitations related to empathy, unexpected clinical scenarios, ethical dilemmas, and professional accountability. These findings suggest that AI should be framed as a complementary tool that supports, rather than replaces, the dentist. Preparing future dentists for responsible AI adoption will require structured curricular integration, hands‐on exposure, critical appraisal skills, and clear guidance on ethical, legal, and patient‐centered use.

This study has several limitations. First, the cross‐sectional design precludes causal inference and does not allow the assessment of changes in AI readiness over time. Second, the study relied on self‐reported questionnaire responses, which may be affected by recall bias, social desirability bias, or overestimation of knowledge. Third, because 62 questionnaires were excluded for incomplete or incorrect responses, nonresponse or exclusion bias cannot be ruled out, as excluded students may have differed from included participants in their AI knowledge, interest, or use. Fourth, the use of convenience sampling and the focus on senior dental students in Erbil may limit the generalizability of the findings to other regions or educational settings. Fifth, although composite scores were useful for summarizing readiness, some domains, particularly the knowledge/use index, comprised heterogeneous exposure‐related indicators and should be interpreted descriptively. Finally, students’ perceptions may change as AI tools become more available in dental education and clinical practice. Future multicenter and longitudinal studies are recommended to evaluate changes in AI readiness and the impact of structured AI education over time.

## 5. Conclusion

Senior dental students in this study showed generally favorable readiness toward AI in dentistry, particularly for diagnostic applications, treatment planning, implant planning, and quality control. Students recognized AI’s potential to improve efficiency and accuracy, but they also expressed concerns regarding empathy, professional accountability, ethical issues, and the need for human oversight. Most participants supported the inclusion of AI‐related topics in dental education; however, given the cross‐sectional design and the single‐setting nature of the study, this finding should be interpreted cautiously. Structured AI education may be considered as a potential strategy to improve students’ knowledge and preparedness, but further multicenter and longitudinal studies are needed before making broad curriculum recommendations. Overall, the findings suggest that students view AI as a complementary tool that may support, but not replace, the clinical judgment and human‐centered aspects of dental care.

## Author Contributions

**Nasreen Hamarash Hamonari**: conceptualization, methodology, investigation, data curation, formal analysis, writing – original draft, writing – review and editing, project administration, supervision, manuscript guarantor.

## Funding

This study did not receive any specific funding.

## Disclosure

This study was reported in accordance with the STROBE guidelines for cross‐sectional studies. The author has read and approved the final version of the manuscript. Nasreen Hamarash Hamonari, as the corresponding author and manuscript guarantor, had full access to all data in this study and takes full responsibility for the integrity and accuracy of the data and analysis.

## Consent

Informed consent was obtained from all participants before participation in the study.

## Conflicts of Interest

The author declares no conflicts of interest.

## Data Availability

The data supporting the findings of this study are available from the corresponding author upon reasonable request.
